# Case Report: Lower Limb Muscle Weakness in a Child With Kawasaki Disease

**DOI:** 10.3389/fped.2022.893568

**Published:** 2022-06-21

**Authors:** Lilin Huang, Shumei Peng, Jing Li, Danyu Xie

**Affiliations:** Department of Pediatrics, Guangdong Women and Children Hospital, Guangzhou, China

**Keywords:** Kawasaki disease, muscle weakness, myositis, systemic vasculitis, child

## Abstract

Kawasaki disease (KD) is a systemic vasculitis that may impact multiple organ systems in children. Myositis is an unusual presentation of KD that presents with muscle weakness. To date, a few pediatric patients with KD and myositis have been reported. Diffuse muscle weakness involving the 4 limbs was the most common presentation in these children. However, isolated lower limb involvement was rarely reported before. Here, we report lower limb muscle weakness in an 18-month-old child with KD. He presented with fever, rash, conjunctival injection, peeling over fingers and toes, and progressive muscle weakness of the lower limbs. Muscle enzymes were normal, but electromyography indicated myositis. The symptom of fever was relieved quickly by intravenous immunoglobulin and aspirin, which were ineffective for myositis. However, lower limb muscle weakness fully recovered 5 days after prednisolone treatment. This rare case might add value to the growing literature exploring the association of KD with myositis.

## Introduction

Kawasaki disease (KD) is a systemic vasculitis with unknown etiology that commonly affects children under 5 years old (1). The principal clinical feature of KD includes polymorphous rash, oral mucosal changes (red and cracking of lips, “strawberry tongue”), bilateral non-purulent conjunctival injection, extremity changes (erythema and edema of the hands and feet in the acute phase, Periungual peeling in the subacute phase), and cervical lymphadenopathy (≥1.5-cm diameter, usually unilateral). Patients with persistent fever (≥5 days) that fulfill ≥ 4 of the five principal clinical features are diagnosed with typical KD (1). Incomplete KD is considered when patients present with less than 4 out of 5 principal clinical features. Patients with atypical KD often involved multiorgan impairment, such as kidney, central nervous system, and muscle (2). The diagnosis of KD is based on the clinical presentation without confirmatory laboratory tests for this disease. KD may impact multiple organ systems due to the systemic inflammatory response. Therefore, KD is an exceptional disease when it presents with unusual presentations (2). Incomplete forms or atypical presentations of KD may create a conundrum of diagnosis and delay therapeutic decision-making for pediatricians (2). To date, a few pediatric patients of KD with myositis have been reported. Myositis is an unusual presentation of KD that presented with muscle weakness, leading to difficulty walking, respiratory failure, eyelid ptosis, dysphagia, and dysphonia in previous studies (3–11). Diffuse muscle weakness involving the 4 limbs was the most common presentation in these children. However, isolated lower limb involvement was rarely reported before. The objective of the study was to report an atypical KD case with lower limb muscle weakness that was resolved by prednisolone within 5 days. Speculation of the potential mechanism of myositis in KD was also discussed.

## Case Report

**FIGURE 1:**
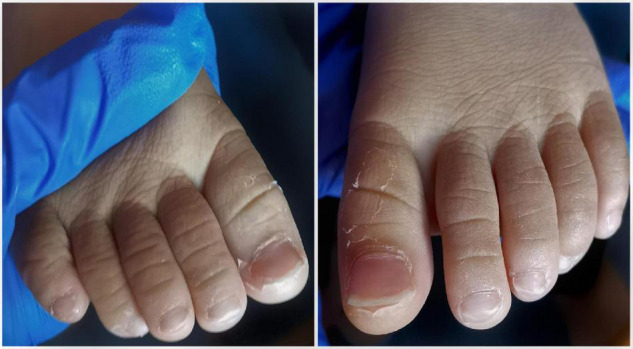
Periungual peeling of skin in both feet.

An 18-month-old boy was admitted to the first hospital with a history of fever for 6 days. On Day 4 of illness, he developed an erythematous rash and muscle weakness of the lower limbs, which presented him with difficulty walking. The muscle strength of the right limb was worse than that of the left limb. On Day 6 of the illness, a non-purulent conjunctival injection appeared. Laboratory investigations showed white blood cell, 20.79 × 10^9^/L; neutrophils, 70.9%; hemoglobin, 95 g/L; lymphocyte, 23%; platelet, 460 × 10^9^/L; procalcitonin, 0.29 ng/ml; and C-reactive protein, 59.94 mg/L. The muscle enzymes were normal. A clinical possibility of KD with neuropathy or arthritis was initially considered. He was treated with intravenous immunoglobulin (IVIG) 2 g/kg, and aspirin, 30 mg/kg/day. The symptoms of fever, rash, and conjunctival injection were relieved. He developed progressive lower limb muscle weakness, leading to difficulty in getting up by Day 10 of the illness. On Day 11 of the illness, the child was brought to us with complaints of persistent muscle weakness without pain. On examination, the muscle strength of the left lower limb was scaled as Grade III, and that of the right lower limb was scaled as Grades II-III. Repeated blood tests showed thrombocytosis (platelet, 698 × 10^9^/L) and an increased erythrocyte sedimentation rate (ESR, 106 mm/h), but creatine kinase (CK), lactate dehydrogenase (LDH), and ferritin were normal by Day 12 of the illness. The child was not exposed to any potential contact with patients affected with COVID-19, and the SARS-CoV-2 reverse transcription-polymerase chain reaction (RT-PCR) test of the nasopharyngeal swab was negative. Serological IgM tests for influenza virus, parainfluenza virus, adenovirus, EB virus, and *Mycoplasma pneumoniae* were all negative. Echocardiography showed that the coronary artery was normal. Electromyography (EMG) indicated myositis. Lumbar puncture was performed, and cerebrospinal fluid (CSF) analysis was normal. Oligoclonal zone, antibodies of myelin basic protein, aquaporin-4, myelin oligodendrocyte glycoprotein, *N*-Methyl-d-aspartate receptor, anti-α-amino-3-hydroxy-5-methyl-4-isoxazolepropionic acid 1/2, contactin-associated protein-like 2, leucine-rich glioma-inactivated 1, gamma-aminobutyric acid receptor in CSF were all negative. The results of cerebral and full-spine MRI were normal. An acetylcholine receptor antibody in serum was negative. On Day 21 of the illness, the child developed pallor and periungual peeling of skin in the fingers and toes (Figure 1). Therefore, atypical KD with myositis was considered. Considering the progressive muscle weakness even though IVIG and aspirin were given, he was treated with oral prednisolone (1 mg/kg/day). He showed rapid improvement in muscle weakness and was willing to walk 3 days after prednisolone treatment. The patient was discharged, and the muscle weakness completely recovered 5 days after prednisolone treatment. A tapering dose of oral prednisolone (0.5 mg/kg/day) was administered for another 7 days. Repeated echocardiography at 6 weeks of follow-up revealed a normal coronary artery. He remained clinically well on follow-up at 6 months (Table 1).

**TABLE 1:** The timeline of events.

Timeline	Symptoms and signs	Laboratory parameters	Treatment
1–3 days	Fever	WBC 20.79 × 10^9^/L, neutrophils 70.9%, lymphocyte 34.6%, HB 114 g/L, CRP 17.44 mg/L	Oral antibiotics
4–5 days	Fever, erythematous rash, and lower limb muscle weakness	WBC 19.36 × 10^9^/L, neutrophils 66.9%, lymphocyte 23%, HB 99 g/L, PLT 377 × 10^9^/L, CRP 48.2 mg/L, CK was normal	Ceftazidime
6–7 days	Fever, rash, low limb muscle weakness, non-purulent conjunctival injection	WBC 20.79 × 10^9^/L, neutrophils 70.9%, lymphocyte 23%, HB 95 g/L, PLT 460 × 10^9^/L, PCT 0.29 ng/mL, CRP 59.94 mg/L, D-dimer 1.72 mg/L, CK and ferritin were normal	Ceftazidime, IVIG 2 g/kg, and aspirin 30 mg/kg/day
8–10 days	The symptoms of fever, rash, and conjunctival injection were relieved. Lower limb muscle weakness progressed	Coronary artery was normal	Aspirin 30 mg/kg/day
11–18 days	Lower limb muscle weakness resulted in difficulty in getting up	WBC 12.43 × 10^9^/L, neutrophils 40.2%, lymphocyte 50.9%, HB 92 g/L, PLT 698 × 10^9^/L, CRP 12.88 mg/L, ESR 106 mm/h, PCT 0.29 ng/mL, CK 48 U/L, CK-MB 24 U/L, Ferritin 67.46 ng/mL, cerebral MRI and CSF analysis were normal, EMG indicated myositis	Aspirin 5 mg/kg/day
19–21 days	Lower limb muscle weakness resulted in difficulty in getting up	Coronary artery was normal	Aspirin 5 mg/kg/day, prednisolone 1 mg/kg/day
22nd day	Improvement in muscle weakness. The child was willing to walk	-	Discharge with aspirin and prednisolone
6 months later	Clinically well	Coronary artery was normal	-

*WBC, white blood cell; HB, hemoglobin; PLT, platelet; CRP, C-reactive protein; ESR, erythrocyte sedimentation rate; CK, creatine kinase; CK-MB, creatine kinase MB; MRI, magnetic resonance imaging; CSF, cerebrospinal fluid; EMG, electromyography; IVIG, intravenous immunoglobulin.*

## Discussion

The KD might involve inflammation in various systems, which requires early diagnosis and prompt treatment to prevent coronary artery injury (12). Myositis is a rare presentation of KD, leading to muscle weakness (2). There have been a few cases with the co-occurrence of KD and myositis. However, isolated lower limb involvement was rarely reported. We reported a child with atypical KD, who showed lower limb muscle weakness. A clinical possibility of juvenile idiopathic arthritis or Guillain–Barre Syndrome was also considered initially since arthritis, arthralgia, myalgia, and neuropathy could also make it difficult for patients to walk. Children under 5 years old might be too young to express the exact symptoms, which could lead to misdiagnosis, especially when muscle enzymes are normal. However, fever was relieved quickly by IVIG, and the sign of peeling over fingers and toes was found on Day 21 of the illness, suggesting the possible diagnosis of atypical KD or multisystem inflammatory syndrome in children (MIS-C) with myositis in our case. MIS-C association with SARS-CoV-2 infection has drawn great attention during the COVID-19 pandemic (13–16). Children with MIS-C might test positive for SARS-CoV-2 by RT-PCR or antibodies in serum since they might be exposed to potential contact with a household member affected with coronavirus disease 2019 (COVID-19) (17, 18). MIS-C might present with KD-like signs and symptoms with more prone to develop toxic shock syndrome (TSS)/macrophage activation syndrome (MAS), resulting in multiorgan failure (19–22). Gastrointestinal and cardiovascular systems are the most affected (14, 17, 23, 24). Children with MIS-C are often older than 5 years of age (18). Laboratory tests might show thrombocytopenia, the elevation of CRP, ferritin, troponin, and D-dimer in patients with MIS-C (25, 26). Our case showed symptoms of fever, skin rash, conjunctival injection, and periungual peeling of skin in the fingers and toes, which could be found in KD and MIS-C. However, the child was 18 months old without the symptoms of abdominal pain, diarrhea, and myocarditis. Laboratory tests showed thrombocytosis with a normal level of total lymphocyte counts, ferritin, CK-MB, and D-dimer. SARS-CoV-2 RT-PCR test of the nasopharyngeal swab was performed three times, which all showed negative results. In addition, the child was not exposed to any potential contact with patients affected with COVID-19. Meanwhile, people were not affected by COVID-19 after contact with this child. Therefore, KD but not MIS-C was diagnosed in our case.

We reviewed previous cases of KD with myositis (Table 2). The age of these cases ranged from 8 months to 10 years, and the timing of muscle weakness that developed in KD ranged from 36 h to 35 days after the onset. Our case was 18 months old, and he developed muscle weakness on Day 4 of the illness, which was consistent with previous reports. Difficulty walking was the most common symptom. Severe myositis could induce respiratory failure, which requires ventilation support. The association of myositis with coronary artery lesions in KD was uncertain since myositis is a rare presentation of KD. To date, coronary artery injury in KD with myositis has been reported in 4 of 10 cases (including our case), which was higher than the 15–25% of untreated patients (2). This might be related to the following reasons: First, patients with myositis might be associated with a latent exaggerated immune response, which results in immune-complex deposits in multiple organs, including the coronary artery. Second, half of the cases with myositis showed atypical presentation or incomplete form, which might induce a delay in the diagnosis and treatment of KD. Finally, the speculation is based on a review of only 10 cases that ask for more data to draw a confirmed conclusion.

**TABLE 2:** Review of previously reported cases with Kawasaki disease and myositis.

Patient no. (References)	Age/Sex	Distribution of muscle weakness	CK (U/L)	Treatment	Recovery of muscle weakness after treatment	Coronary artery abnormalities	EMG/Muscle biopsy
1 (3)	18 month/F	Proximal muscle, dysphonia, and dysphagia	72	Aspirin 100 mg/kg/day	3 months	NA	EMG indicated myositis
2 (4)	3 year/M	Diffuse muscle of all extremities, being greater proximal than distal	152	Oral salicylates 50 mg/kg/day	2 months	Coronary artery aneurysm	EMG showed myositis, muscle biopsy revealed distortion of fascicular architecture with type 2 fiber atrophy, inflammatory cell infiltration
3 (5)	8 year/M	Diffuse muscle of all extremities	2657	Aspirin 100 mg/kg/day, IVIG 400 mg/kg/day	Over 2 months	Coronary artery aneurysm	EMG indicated myositis, muscle biopsy showed type IIB atrophy and muscle fiber degeneration
4 (6)	8 month/M	Left orbicularis muscle and superior rectus-levator palpebrae complex	NA	Oral salicylate 100 mg/kg/day, IVIG, 2 g/kg methylprednisolone	12 months	Coronary artery aneurysm	Muscle biopsy showed areas of panarteritis and a focus of myositis
5 (7)	6 year/F	Left calf	Normal	Hydrocortisone, IVIG, aspirin, incision, and drainage for left calf	7 days	Normal	Muscle biopsy revealed mild chronic inflammation
6 (8)	10 year/F	Hip extensors	Normal	IVIG 2 g/kg, aspirin, prednisolone 2 mg/kg/day	8–10 weeks	Normal	NA
7 (9)	7 year/NA	Left iliopsoas	NA	IVIG, acetyl salicylic acid, methylprednisolone 2 mg/kg/day, infliximab	1 month	Coronary artery aneurysm	NA
8 (10)	10 year/M	Neck flexor, proximal muscle of all extremities	844	IVIG 2 g/kg, methyl prednisolone 30 mg/kg/day for 3 days and then prednisolone, aspirin	7 days	Normal	NA
9 (11)	3 year/M	Eyelid ptosis, muscle of all extremities	62	IVIG 2 g/kg, aspirin 30 mg/kg/d, methylprednisolone 2.8 mg/kg/d	14 days	Normal	EMG indicated normal
10 (present case)	18 month/M	Lower limb muscle	48	IVIG 2 g/kg, aspirin 30 mg/kg/day, prednisolone 1 mg/kg/day	18 days	Normal	EMG indicated myositis

*CK, creatine kinase; EMG, electromyography; IVIG, intravenous immunoglobulin; NA, not available.*

The underlying mechanism of KD with myositis has not been elucidated since this complication is rare, and little literature focuses on this subject. KD is a multisystem inflammation that affects multiple organs with elevated inflammatory parameters, including ESR, CRP, and procalcitonin (2, 27). Exaggeration of the immune response plays important role in the pathogenesis of multisystem inflammation. Therefore, KD might develop into life-threatening KD shock syndrome or MAS in some children, which is a characteristic of a hyperinflammatory response (22). MIS-C shares overlapping manifestations with KD, which also induces neurologic manifestations and myositis (28, 29). Therefore, myositis might be one of the presentations of hyperinflammation. It seemed that elevated muscle enzymes were not necessary for myositis in KD. Only 3 of 9 patients in previous studies showed elevated muscle enzymes, while the patient who had the highest CK level showed respiratory failure. Except for muscle enzymes, EMG, muscle biopsy, and imaging of muscles were also performed to diagnose myositis in the previous cases (10). Muscle biopsy is the gold standard for the diagnosis of myositis. However, it is not practical in routine clinical work since it is an invasive test. Muscle biopsies were initially performed on 3 patients and revealed type 2 fiber atrophy with or without vasculitis (4–6). MRI was performed on one patient, which showed hyperintensity on T2-weighted images in the premuscular and intermuscular fascia in the leg (7). EMG was performed on 3 patients with muscle weakness of the extremities, who showed myogenic injury (3–5). The muscle enzymes were normal, but EMG revealed the myogenic injury in our case. However, MRI and muscle biopsy were not performed since he showed prompt clinical recovery with steroid treatment, and his parent did not agree with these investigations at that time. We tried to speculate on the potential mechanism of myositis in KD. Limited studies have postulated that immune-complex deposits in affected muscles result in muscle weakness (10). However, the specific antigen and antibody were not revealed in previous studies. There was only one adult patient with KD and neuromuscular abnormalities, who underwent immunofluorescent staining of related muscle. The result showed linear deposition of IgG along the sarcolemma. Meanwhile, the electron microscopy results showed that the muscle structure was well preserved (30). There was no evidence of nerve impairment in our case and in other children with KD and myositis. Muscle weakness without elevated muscle enzymes might indicate muscle fiber apoptosis but not necrosis; therefore, the cell membrane maintained its integrity, and muscle enzymes were not released into the serum. Therefore, muscle atrophy but well-preserved muscle structure was observed. Conversely, elevated muscle enzymes might indicate severe inflammatory infiltration and necrosis of muscles. This could be proven by the previous case, which had severe muscular weakness, leading to respiratory failure associated with the highest CK (5). Therefore, muscle enzymes need to be monitored closely in patients with KD and myositis.

Early treatment with intravenous immunoglobulin and aspirin for KD is recommended by the American Heart Association (12). Corticosteroids have been recommended for IVIG-resistant cases (10). However, there was no consensus on the treatment of KD with myositis. Corticosteroids were used in 5 patients in previous studies. However, the dose and the period of corticosteroids were inconsistent. All patients with muscle involvement had good prognoses with or without corticosteroid treatment. The duration of myositis ranged from 7 days to 12 months for a full recovery. Our case showed progressive muscle weakness even though IVIG and aspirin were used. It seemed that IVIG and aspirin were invalid or showed delayed effects for myositis. Corticosteroids might quickly reduce inflammation and depress the immune response. The dose of prednisolone in our case was smaller than that in previous studies. However, prednisolone showed good effects for myositis, and the child recovered faster than in previous cases. This might be explained by the following reasons: First, myositis might be self-limited inflammation since three cases were also treated with aspirin and/or IVIG, even though they took more time to recover in these cases. Second, myositis in our case was isolated lower limb involvement, which was less severe than in other children. Muscle weakness without elevated muscle enzymes might indicate limited inflammation in the muscle and a good prognosis of myositis in KD.

In conclusion, we report a case of lower limb muscle weakness with KD. Myositis is a rare presentation of KD that might develop during the course. Pediatricians should be vigilant about this unusual complication for the early diagnosis and treatment of KD. There is no consensus on the treatment of myositis in patients with KD. However, corticosteroids might shorten the course of myositis, which has been reported to be fully recovered in all patients. This rare case might extend the knowledge of isolated lower limb involvement in children with KD. Future studies are needed to further explore the mechanism of myositis in KD.

## Data Availability Statement

The original contributions presented in this study are included in the article/supplementary material, further inquiries can be directed to the corresponding author.

## Ethics Statement

Ethical approval was obtained from the Ethics Committee of Guangdong Women and Children Hospital for this study. Written informed consent was obtained from the legal guardian, to participate in this study and for the publication of any potentially identifiable images or data included in this article.

## Author Contributions

LH and SP: study concept and design. JL: acquisition of data. LH and DX: analysis and interpretation of data. LH: drafting of the manuscript. All authors have read and approved the manuscript.

## Conflict of Interest

The authors declare that the research was conducted in the absence of any commercial or financial relationships that could be construed as a potential conflict of interest.

## Publisher’s Note

All claims expressed in this article are solely those of the authors and do not necessarily represent those of their affiliated organizations, or those of the publisher, the editors and the reviewers. Any product that may be evaluated in this article, or claim that may be made by its manufacturer, is not guaranteed or endorsed by the publisher.
